# Cardioprotective Effects of Simvastatin in Doxorubicin-Induced Acute Cardiomyocyte Injury

**DOI:** 10.3390/ijms26199440

**Published:** 2025-09-26

**Authors:** Roberta Vitale, Mariangela Mazzone, Maria Carmela Di Marcantonio, Stefania Marzocco, Gabriella Mincione, Ada Popolo

**Affiliations:** 1 Department of Pharmacy, University of Salerno, 84084 Fisciano, Italy; rvitale@unisa.it (R.V.); smarzocco@unisa.it (S.M.); 2 Department of Innovative Technologies in Medicine and Dentistry, University “G. d’Annunzio” Chieti-Pescara, 66100 Chieti, Italy; mariangelamazzone84@gmail.com (M.M.); dimarcantonio@unich.it (M.C.D.M.)

**Keywords:** doxorubicin, cardiotoxicity, simvastatin, oxidative stress

## Abstract

Oxidative stress and mitochondrial dysfunction play a key role in the early stage of Doxorubicin (Doxo)-induced cardiotoxicity. Our study investigated the potential cardioprotective role of Simvastatin (Sim), widely known for its antioxidant properties, in an in vitro model of Doxo-induced acute cardiotoxicity. Human Cardiomyocytes (HCMs) were treated with Sim (10 µM, 4 h) and then co-exposed to Doxo (1 µM) and Sim for 20 h. Our data showed that Sim co-treatment significantly (*p* < 0.05) reduced both cytosolic and mitochondrial Doxo-induced reactive oxygen species overproduction. In Sim co-treated cells, significant reductions in nuclear factor erythroid 2-related factor 2 (Nrf2) gene expression (*p* < 0.01) and catalase (CAT), heme-oxygenase 1 (HO-1), and superoxide dismutase 2 (SOD2) levels (*p* < 0.05) compared to Doxo-treated cells were also demonstrated, suggesting a decreased need for compensatory antioxidant defense responses. Moreover, significant reductions in Doxo-induced mitochondrial calcium overload, mitochondrial membrane depolarization (*p* < 0.005), and apoptosis (*p* < 0.005) confirmed the protective effects of Sim co-treatment on cardiomyocytes. These data confirm that Sim could be a valuable therapeutic strategy for reducing Doxo-induced HCM damage, preventing the development of dilated cardiomyopathy and long-term heart damage, which are the main limitations of anthracycline use. Finally, real-time PCR analysis revealed that Sim co-treatment significantly reduced (*p* < 0.001) the Doxo-induced overexpression of MAP4K4, a mitogen-activated protein kinase kinase kinase kinase-4 (MAP4K4) involved in oxidative stress-induced cell death, thus suggesting the involvement of other molecular mechanisms in Sim-mediated cardioprotection.

## 1. Introduction

Doxorubicin (Doxo) is a chemotherapeutic drug belonging to the anthracycline family that is widely used in treating several cancers including lymphoma, sarcoma and breast cancer [1]. However, despite significantly increasing cancer-related survival [2], Doxo is associated with severe cardiovascular toxicity that manifests in up to 25% of patients, thus limiting its clinical utility. Doxo-induced cardiotoxicity (DIC) is clinically characterized by reduced left ventricular ejection fraction, increased ventricular wall thickness, arrhythmias and heart failure, with potentially fatal outcomes [3]. An initial classification, based on the time between drug administration and the onset of symptoms, divided DIC into three types: acute cardiotoxicity (after a single dose), early-onset chronic cardiotoxicity (within 1 year), and late-onset chronic cardiotoxicity (5–10 years after) [4,5]. However, recent evidence indicates that Doxo-induced acute and chronic cardiotoxicity are not separate disease entities but rather a potential continuous phenomenon, starting with myocardial cell injury and followed by progressive functional decline, progressively leading to overt heart failure [6]. It is noteworthy that the mechanisms of anticancer action and cardiotoxicity occur through different pathways. Indeed, while the inhibition of DNA topoisomerase II and subsequent DNA damage are responsible for antitumor activity [7], the mechanisms underlying DIC are not well understood. Several studies reported the key roles of oxidative stress, mitochondrial dysfunction, and calcium homeostasis dysregulation and consequent apoptosis in DIC [8,9], since cardiomyocytes are particularly sensitive to elevated reactive oxygen species (ROS) levels and their antioxidant defenses are often already saturated by endogenous oxidative metabolism [10].

Therefore, understanding the mechanisms involved in the early stages of DIC and developing preventive strategies are of paramount importance for ensuring survival and quality of life for patients, regardless of their cancer diagnosis. Currently, dexrazoxane is the only cardioprotective agent approved by the US Food and Drug Administration (FDA) for preventing anthracycline-induced cardiomyopathy, but its use is limited due to the development of secondary malignancies [11]. In recent years, research has focused on identifying potential drugs or optimizing existing pharmacological treatments that can reduce and/or prevent cardiac side effects without compromising the antitumor efficacy of Doxo. Statins, which are 3-hydroxy-3-methylglutatyl coenzyme A (HMG-CoA) reductase inhibitors, are particularly promising in this area because of their pleiotropic biological effects, which range from antioxidant to anti-inflammatory effects, in addition to their cholesterol-lowering activity [12]. Preventive use of statins may be a valuable pharmaceutical approach for mitigating anthracycline-induced cardiotoxicity [13], especially lipophilic statins, such as Simvastatin and Atorvastatin, which more readily cross cell membranes via passive diffusion than hydrophilic statins and show more cholesterol-independent effects in vascular cells and cardiomyocytes [14]. A clinical trial conducted reported that prophylactic use of Atorvastatin may prevent the development of cardiac dysfunction in newly diagnosed breast cancer patients receiving anthracycline-based chemotherapy [15].

In addition, two independent clinical trials are currently underway to evaluate whether combining statin treatment with adjuvant anthracycline therapy can help to preserve normal cardiac function in breast cancer patients over periods of 15 weeks (NCT02096588) and 24 months (NCT01988571), respectively.

Moreover, recent studies showed that statins possess antioxidant activity that can contribute to the attenuation of oxidative stress induced by chemotherapeutic drugs [16].

Based on these observations, this study aimed to investigate whether Simvastatin exerts cardioprotective effects in an in vitro model of Doxo-induced acute cardiotoxicity.

## 2. Results

### 2.1. Effect of Simvastatin Co-Treatment on Doxo-Induced Oxidative Stress

**Figure 1:**
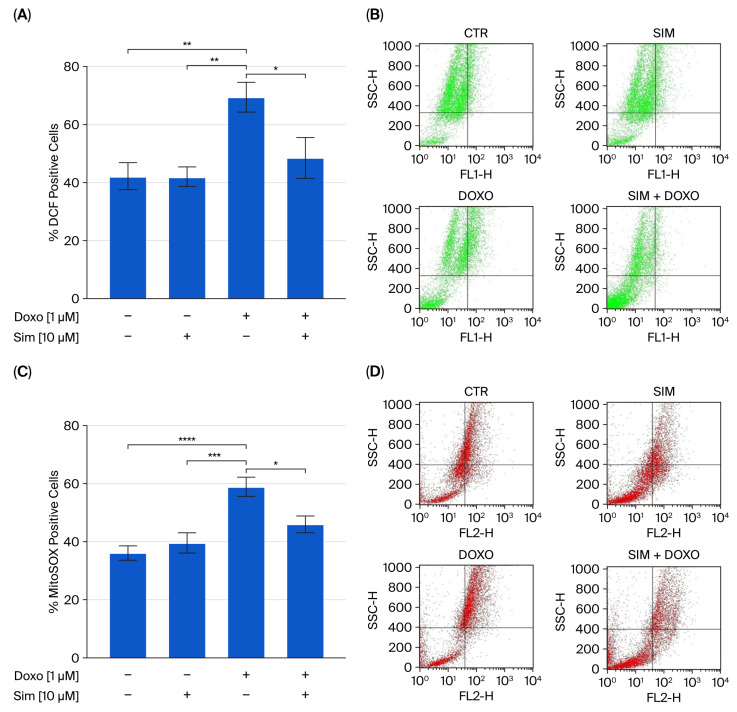
The fluorescent probes 2′-7′dichlorofluorescein diacetate (H_2_DCF-DA) and MitoSOX red, a Mitochondrial Superoxide Indicator, were used to evaluate cytosolic reactive oxygen species and mitochondrial superoxide generation, respectively, via flow cytometry analysis. The results are reported as the mean ± SEM of the percentage of DCF- or MitoSOX-positive cells from at least three independent experiments, each performed in triplicate (*n* = 9). Data were analyzed via variance test analysis, and multiple comparisons were made via Bonferroni’s test. * *p* < 0.05, ** *p* < 0.01, *** *p* < 0.005, and **** *p* < 0.001 (Panels (**A**,**C**)). Representative histograms for the flow cytometry analysis are reported in Panels (**B**) and (**D**), respectively.

Reactive oxygen species (ROS) formation is the most frequently reported and widely recognized primary mechanism of DIC, as the chemical reactivity of Doxorubicin (Doxo) involves a redox cycle that leads to ROS generation [17]. Therefore, we evaluated the effects of Simvastatin (Sim) co-treatment on Doxo-induced ROS overproduction. Cytofluorimetric analysis using 2′,7′-dichlorofluorescin diacetate H_2_DCF-DA and MitoSOX Red confirmed that Doxorubicin (Doxo) significantly increased ROS production (*p* < 0.01 and *p* < 0.001 vs. control cells). Furthermore, our data demonstrated that co-treatment with Sim significantly reduced the Doxo-induced overproduction of both cytosolic and mitochondrial ROS (*p* < 0.05) (Figure 1A,C).

**Figure 2:**
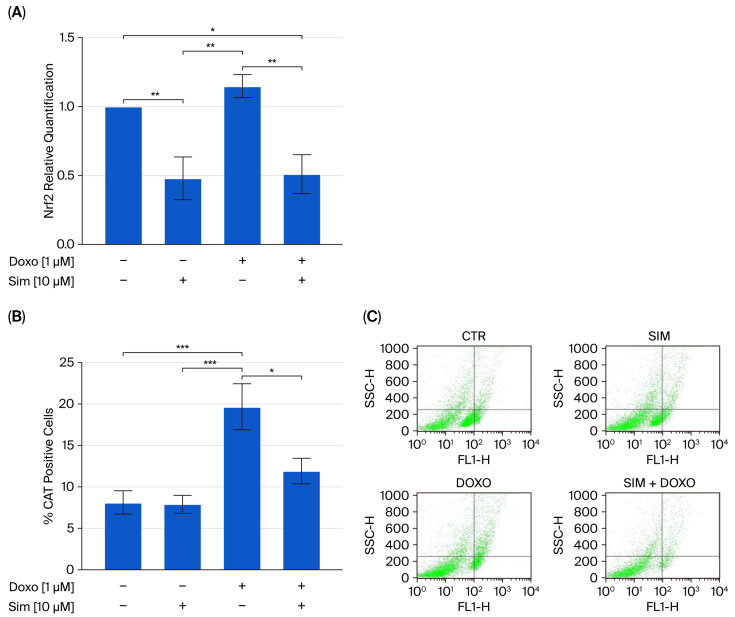

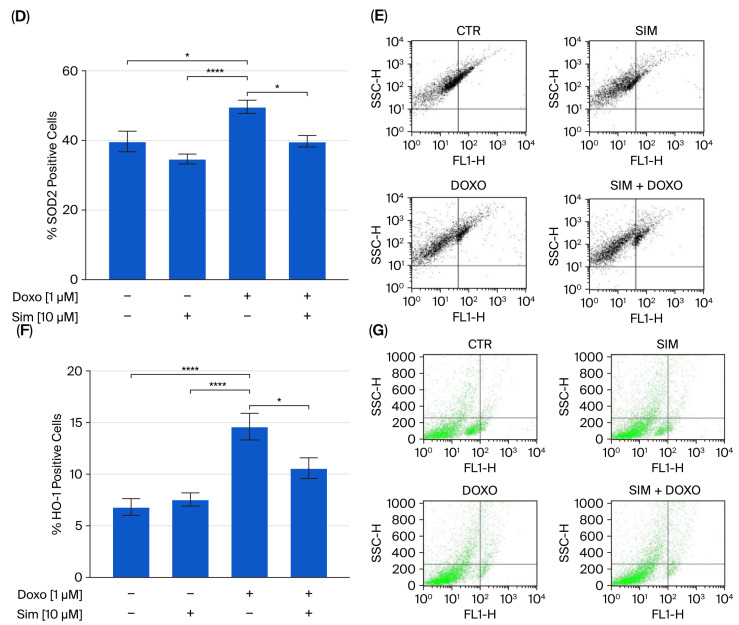
The effects of Sim, Dox, and Sim co-treatment on nuclear factor erythroid 2-related factor 2 (Nrf2) relative gene expression in a Human Cardiomyocyte cell line, as determined via real-time RT PCR. Data were calculated using the 2^−ΔΔCt^ method, normalized to GAPDH cDNA levels and then expressed relative to the control (calibrator sample, defined as 1.00). Values are expressed as means ± SD and were analyzed via analysis of variance (ANOVA), followed by Tukey’s multiple comparisons test. *p < 0.05, ** *p* < 0.01 (Panel (**A**)). To evaluate catalase (CAT), superoxide dismutase 2 (SOD2), and heme-oxygenase 1 (HO-1) levels, flow cytometry analysis was used. Values are expressed as the mean ± SEM of % of CAT-, SOD2-, and HO-1-positive cells from at least three independent experiments, each performed in triplicate (*n* = 9; Panels (**B**,**D**,**F**)). Statistical analysis was performed using one-way ANOVA followed by the Bonferroni multiple comparisons test. * *p* < 0.05, ** *p* < 0.01, *** *p* < 0.005, and **** *p* < 0.001. Representative histograms for the flow cytometry analysis are reported in Panels (**C**,**E**,**G**).

It is well established that, upon the onset of oxidative stress, such as in the early stages of Doxorubicin (Doxo) administration, nuclear factor erythroid 2-related factor 2 (Nrf2) translocates to the nucleus, where it promotes the expression of antioxidant genes and ultimately mitigates oxidative damage [18,19]. Data from real-time RT-PCR confirmed the upregulation of Nrf2 gene expression in Doxo-treated cells in our experimental model. In addition, cytofluorimetric analysis revealed that Doxo treatment significantly increased the levels of antioxidant enzymes, including catalase (CAT; *p* < 0.005), heme oxygenase-1 (HO-1; *p* < 0.001), and superoxide dismutase 2 (SOD2; *p* < 0.05), compared to control cells. Notably, our results showed that co-treatment with Sim significantly reduced Doxo-induced Nrf2 overexpression (*p* < 0.01), as well as the upregulation of antioxidant enzyme levels (*p* < 0.05) (Figure 2).

### 2.2. Effect of Simvastatin Co-Treatment on Intracellular Calcium Signaling and Mitochondrial Membrane Potential (MMP)

**Figure 3:**
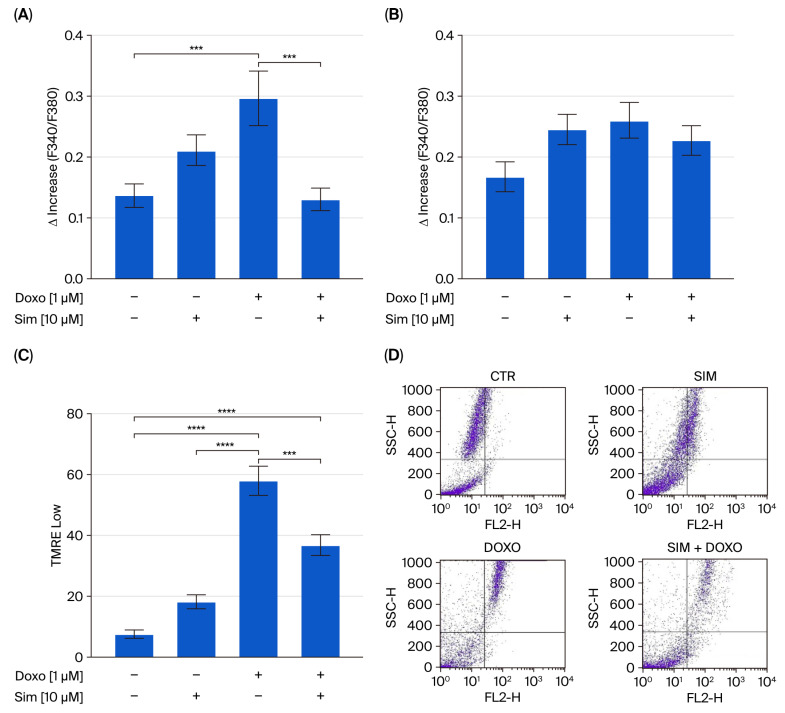
The mitochondrial calcium depletory, carbonyl cyanide p-trifluoromethoxy-pyhenylhydrazone (FCCP, 50 nM) in calcium-free medium was used to evaluate mitochondrial calcium levels (panel (**A**)), and Ionomycin (1 µM) in calcium-free medium was used to evaluate intracellular calcium levels (panel (**B**)) via spectrofluorimetric analysis. The results are reported as mean ± S.E.M. of the percentage of delta increase in FURA2 ratio fluorescence (340/380 nm) from at least three independent experiments, each performed in triplicate. The fluorescent dye tetramethylrhodamine methyl ester (TMRE) was used to evaluate mitochondrial membrane potential via flow cytometry (panel (**C**)). Results are expressed as mean ± SEM of fluorescence intensity of at least three independent experiments each performed in triplicate (*n* = 9). Representative histograms for the flow cytometry analysis are reported in Panel (**D**). The results are expressed as mean ± SEM of fluorescence intensity from at least three independent experiments, each performed in triplicate (*n* = 9). Statistical analysis was performed using one-way ANOVA, followed by the Bonferroni multiple comparisons test. *** *p* < 0.005, **** *p* < 0.001.

The dysregulation of intracellular calcium homeostasis and increased mitochondrial calcium storage with consequent mitochondrial membrane potential depolarization have been well established in DIC [20,21]. Therefore, we analyzed intracellular calcium levels and mitochondrial membrane potential to determine whether Sim co-treatment could also mitigate these Doxo-induced effects in our experimental model. Spectrofluorimetric analysis revealed that Doxo treatment significantly increased mitochondrial calcium accumulation (*p* < 0.005; Figure 3A) without affecting cytosolic calcium levels (Figure 3B). Moreover, cytofluorimetric analysis using the fluorescent dye tetramethylrhodamine methyl ester (TMRE) indicated significant mitochondrial membrane depolarization in Doxo-treated cells (*p* < 0.001; Figure 3C). Notably, Sim co-treatment significantly reversed both Doxo-induced mitochondrial calcium overload and membrane depolarization (*p* < 0.005; Figure 3A,C).

### 2.3. Effect of Simvastatin Co-Treatment on Doxorubicin-Induced Apoptosis

**Figure 4:**
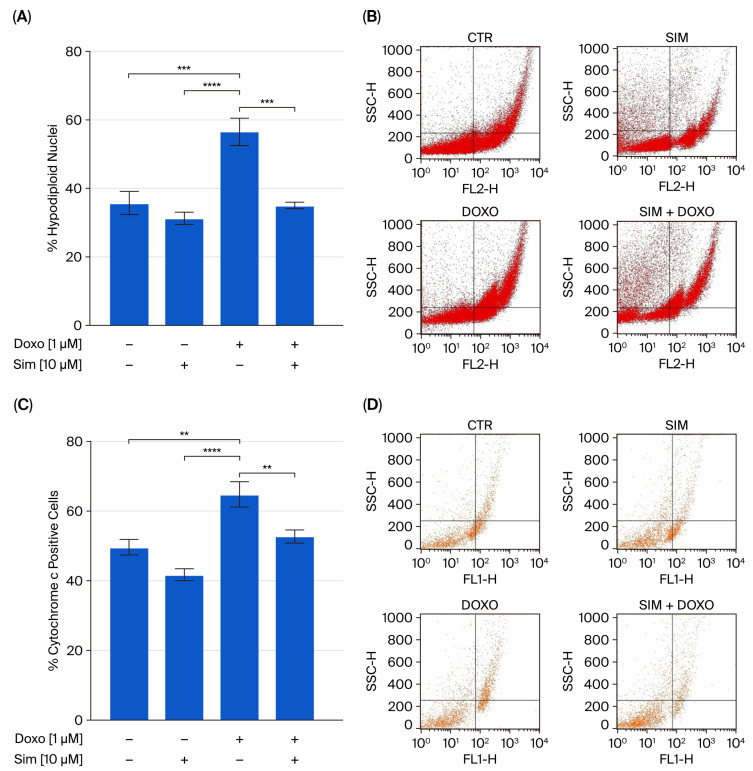
Cells were stained with propidium iodide, and the fluorescence levels of individual nuclei were measured via flow cytometry. The results are reported as the mean ± S.E.M. of the percentage of hypodiploid nuclei from at least three independent experiments, each performed in triplicate (Panel (**A**)). Flow cytometry analysis was used to evaluate the cytosolic cytochrome c level (Panel (**C**)). The results are reported as the mean ± S.E.M. of cytochrome c-positive cells’ percentage from at least three independent experiments, each performed in triplicate (*n* = 9). Representative histograms for flow cytometry analysis are reported in Panels (**B**) and (**D**), respectively. Data were analyzed via variance test analysis, and multiple comparisons were made via Bonferroni’s test. ** *p* < 0.01, *** *p* < 0.005, and **** *p* < 0.001.

It has been well established that oxidative stress plays a significant role in apoptosis, the most common type of programmed cell death in DIC [22]. Indeed, the increase in mitochondrial ROS levels leads to mitochondrial membrane damage, thus inducing apoptosis [23]. Our data showed that Sim co-treatment significantly (*p* < 0.005) reduced Doxo-induced apoptosis, as demonstrated through the cytofluorimetric analysis of hypodiploid nuclei performed using propidium-iodide (Figure 4A). The significant (*p* < 0.01) reduction in cytochrome c release (Figure 4C) confirms the protective role of Sim in our experimental model.

**Figure 5:**
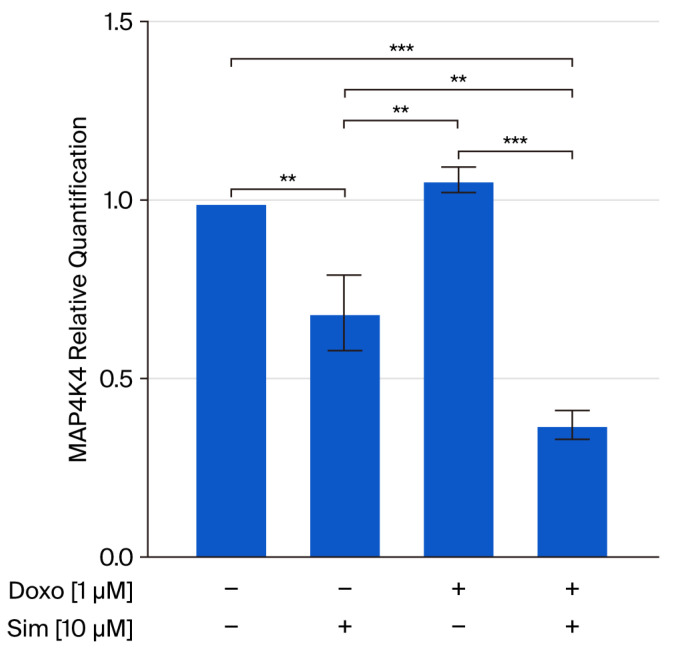
The effects of 24 h Sim, Dox, and Sim co-treatment on mitogen-activated kinase4 kinase kinase kinase-4 (MAP4K4) relative gene expression in a Human Cardiomyocyte cell line, as determined via real-time RT PCR. Data were calculated using the 2^−ΔΔCt^ method, normalized to GAPDH cDNA levels and then expressed relative to the control (calibrator sample, defined as 1.00). Values are expressed as means ± SD and were analyzed via analysis of variance (ANOVA), followed by Tukey’s multiple comparisons test. ** *p* < 0.01, *** *p* < 0.001.

Recent studies showed that mitogen-activated kinase4 kinase kinase kinase-4 (MAP4K4) plays a central role in DIC since it is involved in mitochondrial dysfunction, as well as in cardiomyocyte death [24,25]. Thus, we analyzed MAP4K4 gene expression in our experimental model, and the results obtained showed that Sim co-treatment significantly (*p* < 0.001) reduced Doxo-induced MAP4K4 gene overexpression (Figure 5).

## 3. Discussion

Cardiotoxicity, which is responsible for increased morbidity and mortality in cancer survivors, is recognized as the most important long-term side effect that limits the clinical utility of a drug [26]. Chronic cardiotoxicity, which can occur years after drug discontinuation and clinically manifests as dilated cardiomyopathy and heart failure, has been widely studied over the years by cardio-oncologists, and several pharmacological treatments for use after cancer therapy-related cardiac dysfunction occurs have been recognized [27]. Recently, the scientific community has focused on the study of acute cardiotoxicity, since it has been demonstrated that Doxorubicin-induced cardiotoxicity (DIC) has an early onset, caused by damage to cardiomyocytes induced upon first exposure to the drug. Indeed, it has been shown that several cell death pathways are triggered during Doxorubicin (Doxo) administration, and increasing evidence indicates that oxidative stress, calcium homeostasis impairment, and mitochondrial dysfunction are all involved in DIC. Identifying potential drugs that can reduce and/or prevent Doxo-induced cardiac side effects without affecting its antitumor efficacy is of paramount importance. In this study, we evaluated the cardioprotective effects of Simvastatin (Sim) in an in vitro short time model of DIC, in agreement with the drug repurposing approach that aimed to identify new therapeutic indications for drugs already on the market to reduce the development time and costs. Statins hold promise as protective agents in anticancer treatment-associated cardiotoxicity because of their pleiotropic effects, especially their antioxidant activity. Moreover, several studies proved the cardioprotective effects of Sim in chemotherapeutic drug-induced cardiotoxicity [8,28], and, recently, we showed that Sim enhances the cytotoxic effects of Doxo in cancer cells [29].

Since there is a large discrepancy among the statin concentrations used in various studies, our experimental protocol was defined based on previous reports [8,30]. The MTT assay was performed to confirm that, under our experimental conditions, the selected concentration of Sim (10 µM) reduced Doxo-induced cell death without affecting cell viability when administered alone (Supplementary Materials Figure S1 and Text S1). Moreover, it has been shown that the pleiotropic effects of statins appear at concentrations of 1–50 µM even if, at therapeutic doses, the mean concentration of statins in human serum ranges from 1 to 50 nM [31]. Then, we analyzed the effects of Sim co-treatment on Doxo-induced oxidative stress, mitochondrial dysfunction, calcium homeostasis, and apoptosis. It is well known that Doxo induces the generation of free radicals and activates ROS through redox cycling, a process involving Nicotinamide Adenine Dinucleotide Phosphate (NADP) oxidase and mitochondrial NADH dehydrogenase [32]. Here, we showed that Sim co-treatment significantly reduced Doxo-induced ROS overproduction.

These results are consistent with numerous studies conducted over the years investigating the possible antioxidative activity of statins. The mechanisms via which statins reduce oxidative stress have not been clearly elucidated, but the results gathered suggest that two mechanisms may be involved. In the first case, which mainly involves statins with a lipophilic structure, such as Simvastatin and Atorvastatin, a mechanism involving the direct scavenging of free radicals has been hypothesized. Statins directly neutralize free radicals, thereby protecting cellular structures from oxidative damage [33]. The second hypothesized mechanism involves the inhibition of Rac1 geranylgeranylation. Statins inhibit this key regulator of the NADPH oxidase complex, thereby reducing ROS production [14,34]. Thus, we hypothesize that Sim reduces Doxo-induced oxidative stress both directly and indirectly. Under our experimental conditions, we also found that Sim co-treatment significantly attenuated the Doxo-induced upregulation of Nrf2 gene expression, as well as the increased levels of antioxidant enzymes (CAT, SOD2, and HO-1). At first glance, these findings may contradict the numerous studies investigating therapeutic strategies aimed at counteracting Doxo-induced oxidative stress by enhancing antioxidant enzyme levels through Nrf2 nuclear translocation [17,35,36]. However, it is well established that under pro-oxidant conditions, such as those occurring during the early phase of Doxo administration and reproduced in our experimental model, Nrf2 translocates to the nucleus, thereby inducing the expression of antioxidant enzymes as a compensatory response to oxidative stress [17,35,36]. We, therefore, propose that Sim co-treatment mitigates oxidative stress to such an extent that this adaptive response is no longer required. Moreover, the observed reduction in Doxo-induced HO-1 overexpression further highlights the protective effect of Sim co-treatment in our experimental model. Indeed, although HO-1 is generally considered to be a cardioprotective protein, its upregulation has paradoxically been associated with cardiac damage, since it is involved in heme degradation, resulting in the release of free iron, which can trigger ferroptosis, another cell death pathway involved in Doxo-induced cardiac damage [37].

It is well known that mitochondria are the main target of Doxo, and mitochondrial damage is a property of DIC, referred to as a hallmark, significantly impairing heart function [23]. Previous studies showed that Doxo causes Ca^2+^ overload in mitochondria [38]. Although mitochondrial Ca^2+^ uptake was regarded primarily as a safety device in cases of temporary intracellular Ca^2+^ overload [39], the net accumulation of Ca^2+^ into mitochondria leads to mitochondrial membrane depolarization and mitochondrial swelling with the consequent release of an apoptogenic mitochondrial membrane [21,40]. In agreement with a previous study reporting a direct effect of Sim on mitochondrial functions [41], here, we showed that Sim co-treatment significantly reduced Doxo-induced mitochondrial calcium overload, as well as mitochondrial membrane depolarization. The observed reduction in apoptotic cell death and cytochrome c release confirmed the protective effects of Sim in our experimental model.

Several studies reported that the serine/threonine kinase protein kinase kinase kinase kinase-4 (MAP4K4) plays a pivotal role in DIC, since it is required for oxidative stress-induced cell death [24,42]. Moreover, a recent study showed that the inhibition of MAP4K4 exerts protective effects in Doxo-induced acute cardiotoxicity by reducing apoptosis, thus preserving mitochondrial membrane depolarization and calcium fluctuations, thereby improving cardiomyocyte functions [25].

Although several studies have reported a link between statins and MAPK signaling [43,44], our preliminary data primarily showed that Sim co-treatment significantly reduces Doxo-induced MAP4K4 gene expression.

## 4. Methods

### 4.1. Cell Line and Treatment

Human Cardiomyocyte (HCM) cell lines were purchased from Celprogen (Huissen, The Netherlands; Benelux) and cultured in 100 nm Corning dishes containing Human Cardiomyocyte Cell Culture Complete Growth medium with serum and antibiotics (M36044-15S, Celprogen) in a humidified incubator at 37 °C with 5% CO_2_. Cells were seeded at the density required for each of the experimental analyses. To evaluate the cardioprotective effects of Simvastatin on Doxorubicin-induced cardiomyocytes damage, after 24 h of adhesion, cells were pre-treated with Sim (10 µM, #S6196 Sigma, Milan, Italy) for 4 h and then co-exposed to Sim and Doxo (1 µM, #S-5040420001 Sigma-Italy) for 20 h.

### 4.2. Measurement of Intracellular Reactive Oxygen Species (ROS)

To evaluate cytosolic ROS levels, HCM cells (2 × 10^5^ cells/well into 12-well plate) were treated as described above. After treatment, cells were incubated with PBS containing the probe 2′,7′-dichlorofluorescin diacetate (H_2_DCF-DA; 10 µM, Sigma) for 15 min in the dark at 37 °C. Then, the cells were washed with PBS and collected with staining buffer (PBS containing 2% BSA and 0,1% Sodium Azide) for fluorescence evaluation via flow cytofluorometry. H_2_DCF-DA is a non-fluorescent molecule that passively diffuses into cells, where the acetates are cleaved by intracellular esterases to form H_2_DCF. H_2_DCF is rapidly oxidized to the highly fluorescent DCF in the presence of intracellular ROS. Cell fluorescence was evaluated via fluorescence-activated cell sorting (FACSscan; Becton–Dickinson, Milan, Italy) and analyzed using Cell Quest software (version number 5.2.1). The results are reported as the % of DCF-positive cells.

### 4.3. Detection of Mitochondrial Superoxide Formation

Mitochondrial superoxide production was assessed using MitoSOX Red (Waltham, MA, USA), a fluorogenic probe selectively oxidized by superoxide—but not by other reactive oxygen species—and emitting red fluorescence upon oxidation. HCM cells (2 × 10^5^ cells/well into12-well plate) were treated as previously described. At the end of treatment, MitoSOX Red (2.5 µM) was added for 15 min in the dark at 37 °C; then, cells were harvested with staining buffer (PBS containing 2% BSA and 0.1% Sodium Azide) for fluorescence evaluation via flow cytofluorometry. MitoSOX Red Cell Fluorescence was evaluated via fluorescence-activated cell sorting and analyzed using Cell Quest software. The results are reported as the % of MitoSOX-positive cells.

### 4.4. Analysis of Apoptosis

Apoptosis was analyzed using propidium iodide (PI), a fluorochrome capable of binding cellular DNA content. HCM cells (2 × 10^5^ cells/well into 12-well plate) were treated as previously described. Thereafter, a solution containing PI (50 µg/mL, Sigma), 0.1% Triton X-100, and 0.1% sodium citrate buffer was added, and the plate was incubated at 4 °C for 30 min in the dark. Then, cell nuclei were analyzed via fluorescence-activated cell sorting using Cell Quest software. The results are expressed as the % of hypodiploid nuclei.

### 4.5. Flow Cytometry Analysis

HCM cells (1 × 10^4^ cells/well into 96-well plate) were treated as previously reported. To assess intracellular levels of Cytochrome c (Cyt c, Santa Cruz, Dallas, TX, USA), Catalase (CAT, Santa Cruz), Superoxide dismutase (SOD2, Santa Cruz), or heme-oxygenase (HO-1, Santa Cruz) after the incubation period at 4 °C for 20 min with Fixing buffer (PBS containing 1% BSA, 1% Formaldehyde), a permeabilization buffer (Fixing buffer containing 0.1% TritonX) was added and cells were incubated for 30 min at 4 °C. Thereafter, anti-Cyt c, anti-CAT, anti-SOD2, anti-HO-1 (all 1:250), and appropriate secondary antibody (anti-rabbit FITC antibody or anti-mouse FITC antibody; 1:250) were added at 4 °C for 30 min. Then, the cells were collected with Fixing buffer and the fluorescence was evaluated via fluorescence-activated cell sorting using Cell Quest software. The results are reported as the % of positive cells.

### 4.6. Evaluation of Intracellular Calcium Signaling

Intracellular calcium levels were evaluated via the fluorescent indicator Fura 2-AM (Sigma), the membrane-permeant acetoxymethyl ester form of Fura 2. HCM cells (3 × 10^4^ cells/well into 24-well plates) were treated in the manner previously reported. After treatment, cells were rinsed with phosphate-buffered saline (PBS), 300 µL of Hank’s balanced salt solution (HBSS) containing 5 μM Fura 2-AM was added to each well, and the plate was incubated for 45 min at 37 °C. To remove excess Fura 2-AM, cells were washed with the same buffer and then incubated for 15 min in calcium-free HBSS containing 0.5 mM EGTA to allow for the hydrolysis of Fura 2-AM into its active fluorescent form, Fura 2. The mitochondrial calcium depletory, carbonyl cyanide p-trifluoromethoxy-pyhenylhydrazone (FCCP, 50 nM final concentration), and the calcium ionophore, Ionomycin (1 μM final concentration), were added into each well in calcium-free HBSS/0.5 mM EGTA buffer. Analysis was performed using a spectrofluorometer (Perkin Elmer Multi-mode Microplate reader Enspire 2300); the excitation wavelength was alternated between 340 and 380 nm, and emission fluorescence was recorded at 515 nm, since, as previously reported, the fluorescence intensity ratio of 340/380 nm (F340/F380) is strictly related to intracellular free calcium [45,46]. Data are expressed as the percentage of delta increase in the fluorescence ratio (F340/F380 nm) calculated as follows:

$$\frac{\mathrm{Fluorescnce}\;\mathrm{ratio}\;\mathrm{induced}\;\mathrm{by}\;\mathrm{FCCP}\;\mathrm{or}\;\mathrm{Ionomycin}-\mathrm{basal}\;\mathrm{fluorescence}\;\mathrm{ratio}}{\mathrm{basal}\;\mathrm{fluorescence}\;\mathrm{ratio}}$$

### 4.7. Measurement of Mitochondrial Membrane Depolarization

To evaluate mitochondrial permeability transition pore (mPTP) opening, we used the fluorescent dye tetramethylrhodamine methyl ester (TMRE) that, due to its positive charge, penetrates and accumulates the mitochondria in inverse proportion to the membrane potential. HCM cells (2 × 10^5^ cells/well into 12 well plate) were treated as described above, and then the fluorescent dye tetramethylrhodamine methyl ester (TMRE, 5 nM) was added, and the plate was centrifuged at 1500 rpm for 5 min. Thereafter, cells were collected with staining buffer (PBS containing 0.1% Sodium Azide and 2% BSA). Cell fluorescence was evaluated via fluorescence-activated cell sorting and analyzed using Cell Quest software. Data are expressed as the % of TMRE low, as previously reported [21].

### 4.8. RNA Extraction and Quantitative Real Time RT-PCR

The extraction of total RNA was performed from HCM treated in the manner previously described, using Trifast (EuroClone S.p.A., Pero, MI, Italy) in agreement with the manufacturer protocol. RNA samples were assessed for purity and quantified using a Nanodrop 1000 Spectrophotometer (Applied Biosystems, Thermo Fisher Scientific, Waltham, MA, USA). Reference and targets genes were amplified in a volume of 10 μL containing 1 μL of cDNA at a diluted concentration, 0.2 μL of a primer mixture, and 5 μL of the GoTaq^®^ 2-Step RT-qPCR System (Promega) according to the manufacturer’s instructions. The cDNA levels were evaluated using IDT primers (Integrated DNA Technologies, Leuven, Belgium) via SYBR green quantitative real-time RT-PCR (qRT-PCR) analysis using the StepOne^TM^ 2.0 Real-Time PCR system (Applied Biosystems). The cycling conditions were defined as follows: 10 min at 95 °C and 40 cycles of 15 s at 95 °C, followed by 1 min at 60 °C and final elongation of 15 s at 95 °C. Data were analyzed using the comparative Ct method and graphically indicated as 2^−∆∆Ct^ ± SD. In accordance with the method, the cDNA amounts of the target genes were normalized via the ratio of the median value of the endogenous housekeeping, GAPDH, obtained in treated cells vs. untreated cells.

For each experimental condition, the expression of target genes (Table 1) was assessed by performing three replicates in three independent experiments.

### 4.9. Statistical Analysis

Statistical analysis was performed using GraphPad Prism8 (GraphPad Software Inc., San Diego, CA, USA). Data are reported as the mean ± S.E.M. for at least three independent experiments, each performed in triplicate. Statistical analysis was performed via one-way analysis of variance (ANOVA), followed by the Bonferroni’s multiple comparisons test for cytofluorimetric analysis and Tukey’s multiple comparisons test for qRT-PCR analysis. A *p*-value lower than 0.05 was considered statistically significant.

## 5. Conclusions

Overall, our findings support the hypothesis that Simvastatin can effectively mitigate the acute cardiotoxic effects of Doxorubicin. Using an in vitro model, we demonstrated that Simvastatin co-treatment reduces oxidative stress, improves mitochondrial function, prevents calcium overload, and limits the activation of apoptotic pathways induced by Doxorubicin in human cardiomyocytes. Notably, we observed that Simvastatin significantly downregulates the gene expression of MAP4K4, a kinase implicated in oxidative stress-induced cardiac injury. Although our data are limited to mRNA expression, this finding raises the possibility that Simvastatin may exert some of its cardioprotective effects through the modulation of MAP4K4, a hypothesis that warrants further investigation at the protein and functional levels.

These results strengthen the rationale for repurposing Simvastatin, a widely used and well-tolerated drug, as a cardioprotective agent in the context of Doxorubicin-based chemotherapy. Given its pleiotropic properties, particularly its antioxidant activity, Simvastatin may offer a promising therapeutic strategy for preventing chemotherapy-associated cardiac dysfunction without compromising antitumor efficacy. However, further in vivo studies and clinical trials will be necessary to validate these findings and establish their translational potential.

## Figures and Tables

**Table 1:** Primer sequences for reference and target genes. The table lists the sequences of primers used for qRT-PCR assays. Each primer pair was validated for optimal amplification conditions and specificity to the intended target sequence.

Genes	Forward Primer Sequence (5′–3′)	Reverse Primer Sequence (5′–3′)
GAPDH	CAACTTTGGTATCGTGGAAGGAC	ACAGTCTTCTGGGTGGCAGTG
NRF2	CCTGGGATTTATAGCAGCAGAC	TGACACCAACCAGAGCTGAG
MAP4K4	GTTAAAACGGGTCAGTTGGC	CCCCACAGAACTCCATAACAAG

## Data Availability

The data presented in this study are available on request from the corresponding authors.

## Supplementary Materials

The following supporting information can be downloaded at https://www.mdpi.com/article/10.3390/ijms26199440/s1.
