# Supplementary material for: Cardioprotective Effects of Simvastatin in Doxorubicin-Induced Acute Cardiomyocyte Injury
Source: Int J Mol Sci. 2025 Sep 26;26(19):9440. doi: 10.3390/ijms26199440 (PMC12525417; doi:10.3390/ijms26199440)
Supplement: Supplementary file 1 [file ijms-26-09440-s001.zip › ijms-3833939-supplementary updated.pdf]

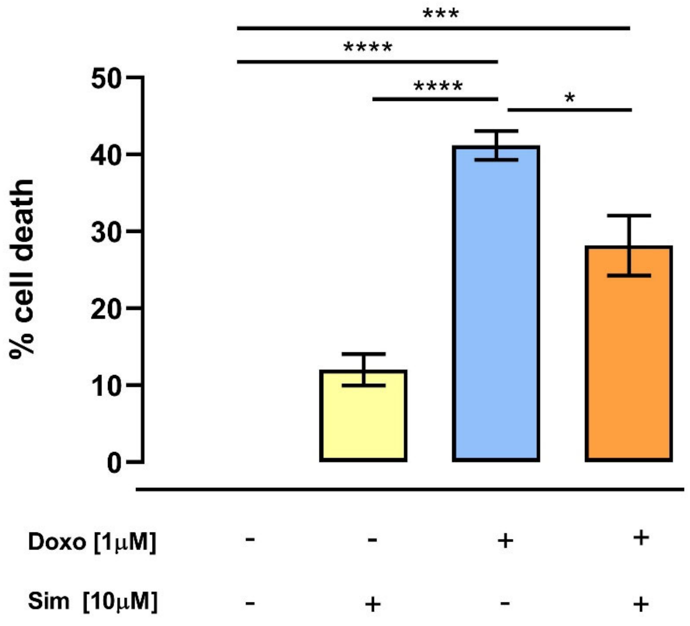

Supplementary Figure S1. Cellular viability was assessed by MTT assay. Cell viability was calculated as % of dead cells = 100 - ([OD treated/ OD control] x 100). Data were analyzed using One-Way ANOVA followed by the Bonferroni multiple comparisons. Values are expressed as mean ± SEM of % cell death. * $p<0.05$ *** $p<0.005$, **** $p<0.001$

Text S1. Materials and Methods

MTT assays

HCM cells ($3.5x10^3$ cells/well into 96 well plate) were pre-treated with Sim for 4h and then co-exposed to Sim and Doxo for 20 h. Cell viability was evaluated by means of 3-(4,5-dimethylthiazol-2-yl)-2,5-diphenyltetrazolium bromide (MTT). At the end of treatment, 25µl of MTT (5mg/ml) were added to each well and plate were incubated for 3 hours to allow the formation of purple formazan precipitate. The solution was then removed from each well and the formazan crystals within cells were solubilized with 100 µl of DMSO. The optical density (OD) of each well was measured with a microplate spectrophotometer (Thermoscientific, Multiskan FC) equipped with a 550 nm filter. Cell mortality was calculated as % of cell death = 100 - [(OD treated/OD control) x 100].
